# Non-equilibrium plasma jet induced thermo-acoustic resistivity imaging for higher contrast and resolution

**DOI:** 10.1038/s41598-017-09964-x

**Published:** 2017-08-25

**Authors:** Liang Guo, Linbo Li, Fanqing Dong, Wencong Jiang

**Affiliations:** College of Information and Control Engineering, China University of Petroleum, Qingdao, Shandong 266580 People’s Republic of China

## Abstract

A thermo-acoustic imaging modality induced by non-equilibrium atmospheric pressure plasma jet is reported. A tiny plasma jet is generated by a fast-rising pulsed dielectric barrier discharge and applied to the surface of the biological tissues. The pulsed conductive current induced by the conductive plasma jet is injected into the biological tissues. The Joule heating inside the tissue stimulates the ultrasound signals effectively. The amplitude of the ultrasound is related to the resistivity of the biological tissues near the contact point and takes the maximum at the certain conductivity of the certain frequency. Accordingly the thermo-acoustic resistivity imaging modality of high contrast and resolution is demonstrated theoretically and experimentally.

## Introduction

High resolution conductivity imaging of biological tissues has received increased attention because of its potential applications in the early diagnosis of the cancer. Magneto-acoustic tomography (MAT) and magnetically mediated thermo-acoustic tomography (MMTAT) are two kinds of conductivity imaging modalities, combining high contrast of conductivities in biological tissue and good resolution of ultrasonography^1–4^. However, because of the weak coupling between electromagnetic excitations and ultrasonic pressures, both imaging modalities have low signal-to-noise ratio (SNR). Only interfaces with different conductivities can produce measurable acoustic signals experimentally. Therefore, in homogeneous conductivity areas, the corresponding conductivity image lacks the internal information^5–8^. If there is an efficient electromagnetic excitation mode, which can generate acoustic signals point by point like photo-acoustic imaging^9^, the signal-to-noise ratio would be greatly improved and the inner conductivity would be reconstructed with higher resolution.

On the other hand, the non-equilibrium atmospheric pressure plasma jet (NEAPPJ) has been introduced to the biomedical applications for many years. The highly active ions in the NEAPPJ exhibit broad application prospects^10–14^. In this paper, a new thermo-acoustic imaging approach induced by the NEAPPJ is investigated. The NEAPPJ is first developed to stimulate the thermo-acoustic signal in biological tissue effectively despite of its wide usages in bacteria inactivation^10^, plasma medicine^11^, wound healing^12^, cancer treatment^13^, and many other biomedical fields^14^.

When the NEAPPJ is stimulated by a fast-rising pulsed voltage, a nanosecond pulsed conductive current is accordingly applied to the tissue through the contact point. A transient Joule heating with high current density near the contact point would generate the thermo-acoustic signal. This signal can be regarded as emitted from the contact point of the tissue, related to its resistivity, and easily detected with high SNR. This approach is immune from the low resolution in homogeneous conductivity areas, providing a more efficient solution in terms of the thermo-acoustic excitation. Because the acoustic impedance of the gaseous plasma jet is far less than that of the biological tissues, another kind of acoustic signal that emitted from the gaseous plasma is seldom transmitted into the tissue. Nearly all the ultrasound that emitted from the gaseous plasma can be reflected back by the interface between the gas and the tissues. Furthermore, because of the flexible contact between the NEAPPJ and the object, the excitation point can be moved randomly on the tissue’s surface. Compared with the traditional conductive electrode, the plasma jet would act as a soft gaseous electrode, scanning at each point to excite thermo-acoustic signals conveniently. The combination of the NEAPPJ and the ultrasonography has the prospect of resistivity imaging with high contrast and resolution for the early diagnosis of the cancer, especially for the cancer on the shallow layer of the tissue. In combine with the NEAPPJ’s application in the cancer treatment^13^, also the resistivity can be detected dynamically to evaluate the treatment effect with the help of the thermo-acoustic imaging.

## Result

Considering the plasma jet with constant conductive current, theoretical analysis shows that the thermo-acoustic signals would take the maximum at certain conductivity value of certain frequency. By considering the displacement current and the complex resistivity, the relationship between the thermo-acoustic signal and the resistivity is described by a detailed deduction.

Under the excitation of the pulsed conductive plasma jet, the pulsed conductive current is injected into the biological tissue. The thermo-acoustic field is excited by the heating effect of this current, which can be represented in the Fourier domain as

1 $${\nabla }^{2}P+{k}^{2}P=-\frac{j\omega \beta }{{C}_{p}}H({\boldsymbol{r}},\omega )$$

where *j*, ***r***, *k*, *ω*, *P*, *β*, *C*_*p*_, and *H* represent the imaginary unit, the point of the field, the wave number, the angular frequency, the thermo-acoustic pressure, the isobaric volume expansion coefficient, specific heat capacity of tissues, and the heat function, respectively. Because the narrow pulsed conductive current has wide bandwidth, the displacement current in the biological tissue could not be ignored. The total current density, including the conductive current density and the displacement current density, can be represented as

2 $${\boldsymbol{J}}=\sigma {\boldsymbol{E}}+j\omega \varepsilon {\boldsymbol{E}}$$

where *σ*, *ε*, ***J*** and ***E*** represent the conductivity, the permittivity, the total current density, and the electric field intensity, respectively. Accordingly, the conductivity or resistivity should be regarded as a complex. Considering the isotropy biological tissues, the heat function *H* in Eq. (1) can be represented as

3 $$H=\mathrm{Re}[\rho ({\boldsymbol{r}})]{|{\boldsymbol{J}}({\boldsymbol{r}},\omega )|}^{2}$$

where Re is the operator for the real part. The *ρ*(***r***) is the complex resistivity located at the point ***r***, which is defined as

4 $$\rho =\frac{\sigma }{{\sigma }^{2}+{\omega }^{2}{\varepsilon }^{2}}-j\frac{\omega \varepsilon }{{\sigma }^{2}+{\omega }^{2}{\varepsilon }^{2}}$$

Because the plasma jet concentrates the current at the contact point, only the current distributed near the contact point would stimulate the measurable acoustic signals with high SNR. Therefore *H* can be simplified as

5 $$H=\frac{\sigma }{{\sigma }^{2}+{\omega }^{2}{\varepsilon }^{2}}{|{\boldsymbol{J}}(\omega )|}^{2}\delta ({\boldsymbol{r}}-{\boldsymbol{r}}\text{'})$$

where ***r***′ represent the contact point, $\delta ({\boldsymbol{r}}-{\boldsymbol{r}}\text{'})$ is the 3-dimension Dirac function denoting the spatial point ***r′*** *.*

Substituting Eq. (5) into Eq. (1), we have

6 $${\nabla }^{2}P+{k}^{2}P=-\frac{j\omega \beta }{{C}_{p}}\frac{\sigma }{{\sigma }^{2}+{\omega }^{2}{\varepsilon }^{2}}{|{\boldsymbol{J}}(\omega )|}^{2}\delta ({\boldsymbol{r}}-{\boldsymbol{r}}^{\prime} )$$

The right part of the Eq. (6), that is, the acoustic source, is proportional to the real part of the complex resistivity without other parameters changed. Under the assumption of homogeneous acoustic medium^8^ the acoustic field can be regarded as a field generated by the point source located at ***r***′, which makes it easy to be reconstructed point by point.

**Figure 1:**
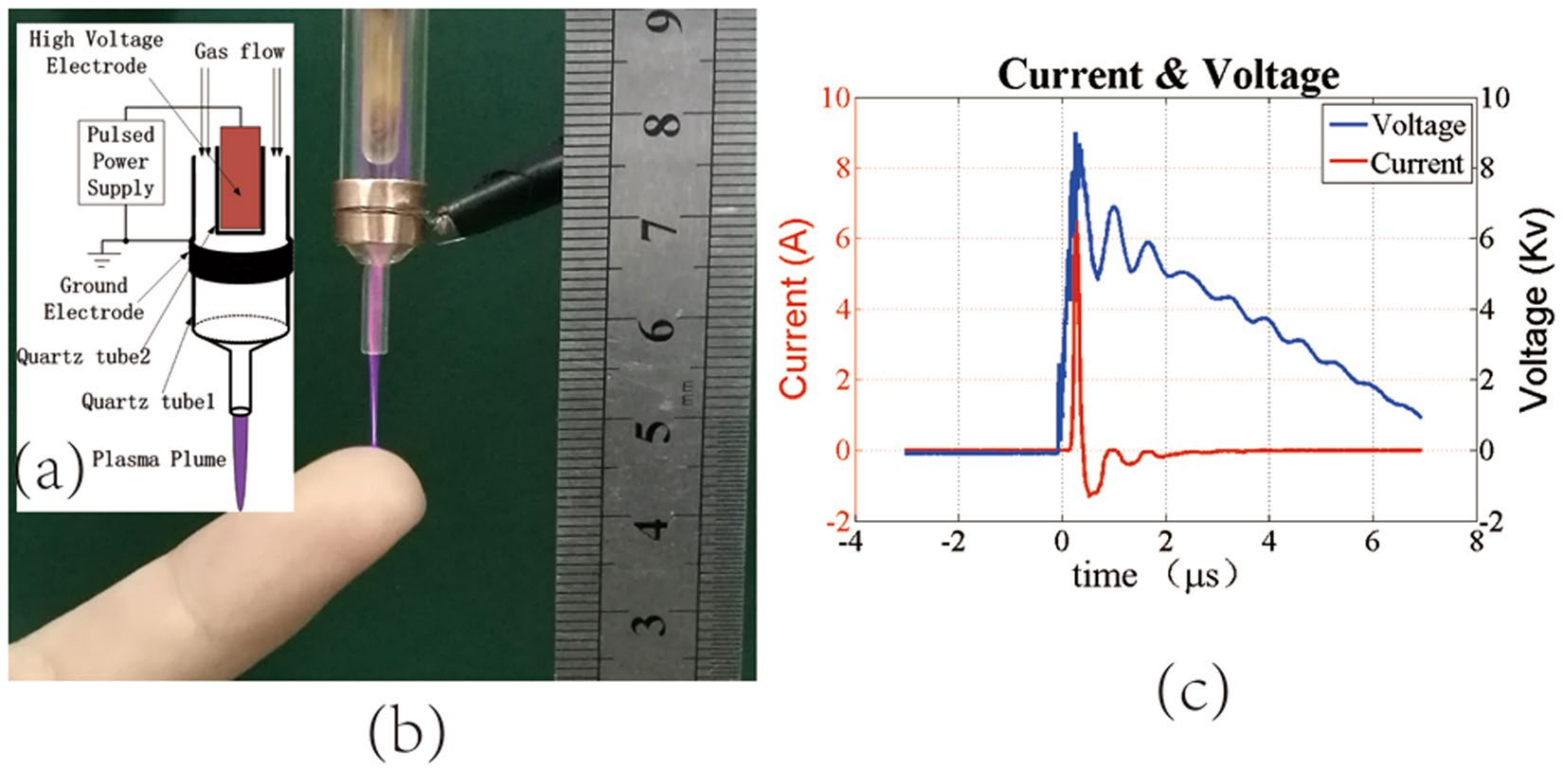
(**a**) Schematic of the NEAPPJ discharge device. (**b**) Photograph of the NEAPPJ contacting with the human finger. (**c**) Total current and excitation voltage.

To improve the electromagnetic excitation under safety condition, the plasma jet has been shaped to a fine needle with a quartz tube and introduced to a safe contact with human bodies^15^. Figure 1(a) and (b) show the schematic of the NEAPPJ discharge device that is customized for our researches and its photograph of contacting with a human finger, respectively. The high voltage (HV) electrode, which is made of a copper wire with a diameter of 2 mm, is inserted into a quartz tube 1 with the bottom end closed. The inner and outer diameters of the quartz tube 1 are 2 mm and 4 mm, respectively. The quartz tube 1 containing the HV electrode is inserted into another quartz tube 2 and centered on the same axis. The inner and outer diameter of the quartz tube 2 is 6 mm and 8 mm, respectively. The ground electrode, which is made of a copper ring, is mounted on the outer surface of the quartz tube 2. The distance between the tip of the inner electrode and the ground electrode is about 5 mm. In order to reduce the diameter of the jet beam, the outlet diameter of the quartz tube 2 is designed to be 0.8 mm. The pulsed high voltage with fast-rising edge is applied to the inner and outer electrodes. When the repeat frequency is set to 200 Hz and the helium is injected from the top of the tube 2 with the flow rate of 4 L/min, the plasma jet is generated with a plume length of 1 cm. It is noticed that the plume can directly contact the human without any feeling of the electrical shock. It can be explained by the main part of the discharge current going through the tube rather than the human bodies. Because there is no other electrode to complete the current loop, the current that is injected into the human body will flow back to the ground in form of the displacement current within the distributed capacity.

In order to quantify the injected current to the target, the current and voltage of the plume are measured by the high-bandwidth probes^16^ connected to a digital oscilloscope. As shown in Fig. 1(c), under the stimulation of the fast-rising voltage, the pulsed conductive current is generated shortly after the rising edge, with the amplitude of 6 A and the time width of 200 ns. It is observed that the conductive currents, which are determined by the ion density of the NEAPPJ, would be constant for each pulse if the length of the NEAPPJ, the voltage, and the flow rate remain unchanged. In fact, if the excitation voltage and the flow rate of the gas remain stable, the ions density and the conductive current would be a variable to the length of the plume. In our experiments, the length of the plume, which is easily to be influenced by the fluctuation of the target surface, is kept uniform by shaping the target with a flat surface.

**Figure 2:**
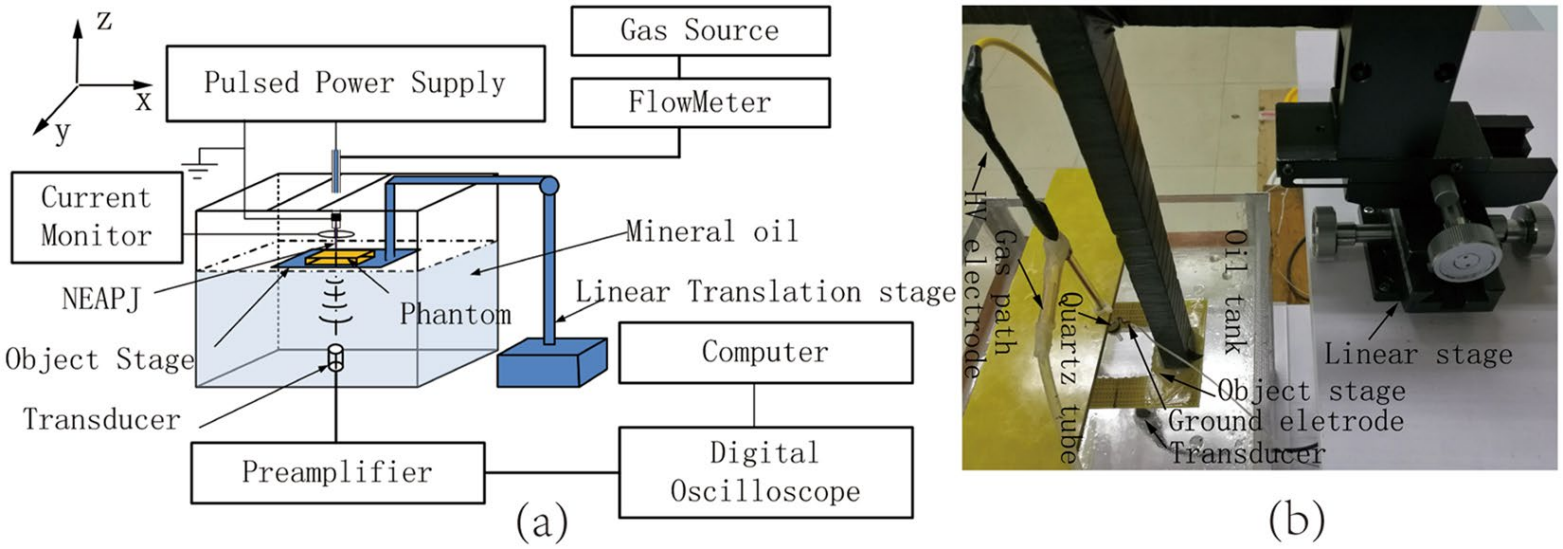
(**a**) Schematic of the experimental setup. (**b**) Photograph of the experimental system.

**Figure 3:**
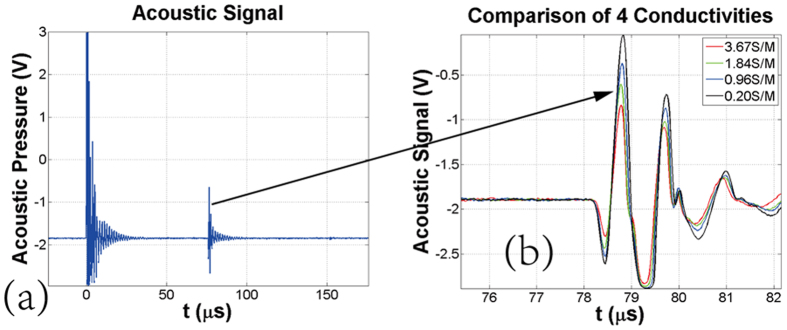
(**a**) Acoustic signals induced by NEAPPJ. (**b**) Comparison of the thermo-acoustic signals of 4 saline-gel phantoms.

To verify the relationships between the acoustic signals and the conductivities, as shown in Fig. 2(a), experiments on well-controlled saline-gel phantoms were first conducted (Described in ‘Method’). Four phantoms with different conductivities of 3.67 S/m, 1.84 S/m, 0.96 S/m and 0.20 S/m (measured by the conductivity meter DDS-307, Shanghai Youke) were made in the same shapes of cylinder. The four phantoms had the same thickness of 5 mm and the diameter of 4 cm. The NEAPPJ was employed to act on the center point of the phantom. Figure 3(a) shows one of the ultrasound signals detected from the phantom of 1.84 S/m. Figure 3(b) shows the details of the thermo-acoustic signals that come from the 4 phantoms. The thermo-acoustic signal yielded a peak at 78.8 μs, agree with the distance of about 11 cm between the contact point and the transducer. Comparing with the MAT and MMTAT, the ultrasound signal’s amplitude reached up to about 1 V with high SNR, which could be quickly detected without thousands of average times. It was also observed that the four thermo-acoustic signals increased as the conductivities decreased. Because the real part of the complex resistivity varied with the excitation frequency, the acoustic signal was not proportional to the resistivity that was measured by the conductivity meter. It was corresponding to the theoretical analysis of the Eq. (6), which indicated that the excitation frequency was one of the influence factors to the thermo-acoustic sources.

**Figure 4:**
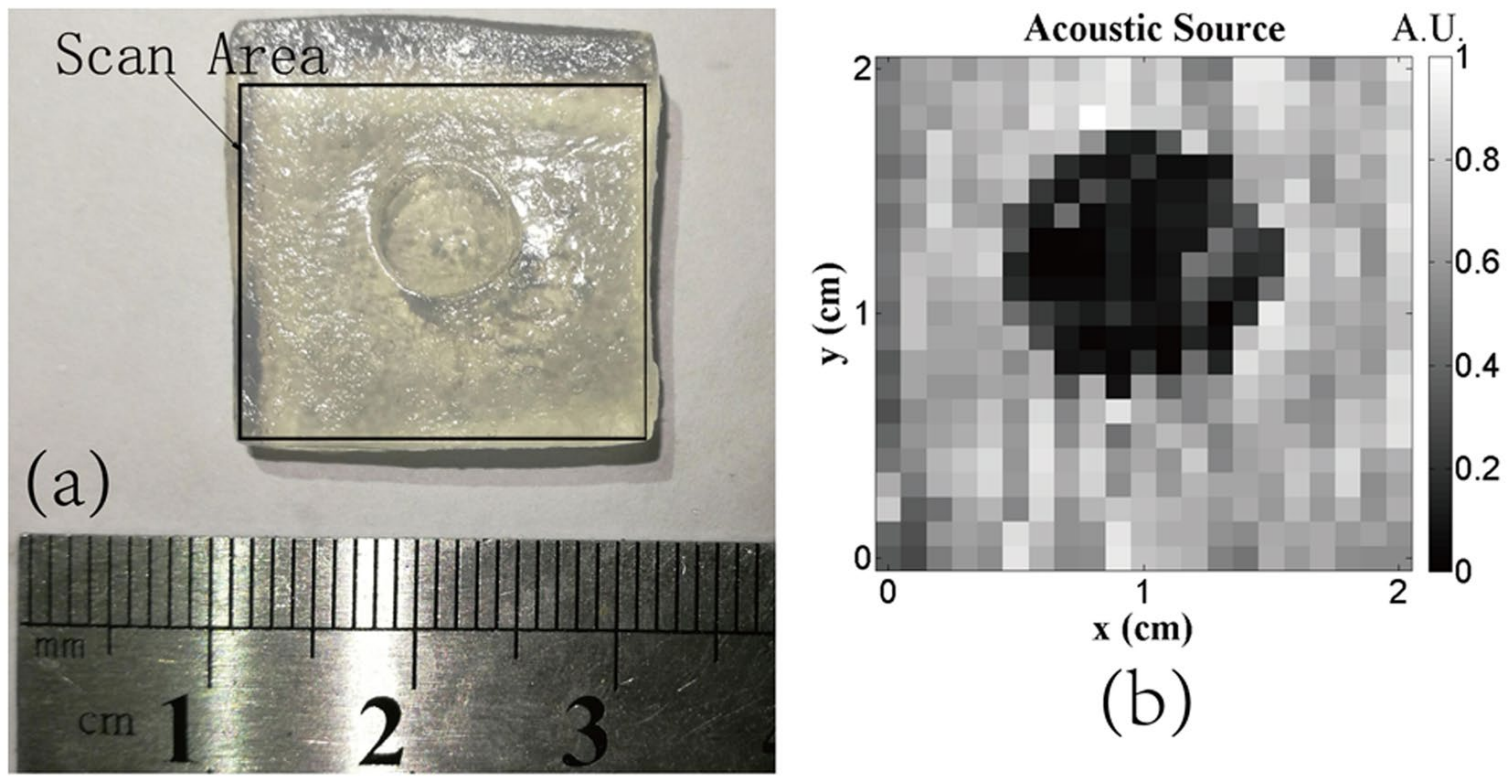
(**a**) Photograph of the saline-gel phantom with two layers. (**b**) Thermo-acoustic source imaging.

Next, the experiment on the saline-gel phantom of double conductivity layers was conducted. Figure 4(a) is the photograph of the saline-gel. The cylindrical inner part of the saline-gel, with the conductivity of 1.84 S/m, was surrounded by the outer saline-gel of 0.96 S/m. The plasma jet scanned the saline-gel phantom by the step length of 1 mm within a square area of 2 × 2 cm^2^, which led to a relative low resolution of 400 pixels just to verify the proposed method. The peak amplitudes of the ultrasounds were directly used to image the thermo-acoustic sources at different scan points of the surface. The thermo-acoustic source image, similar to the resistivity image, was reconstructed as shown in Fig. 4(b). It was observed that the inner cylindrical part could be imaged obviously with the inner homogeneous conductivities. Another interesting result showed that the scale of the inner part was slightly bigger than that of the photograph because of the diffusion effect between the two different conductivities.

**Figure 5:**
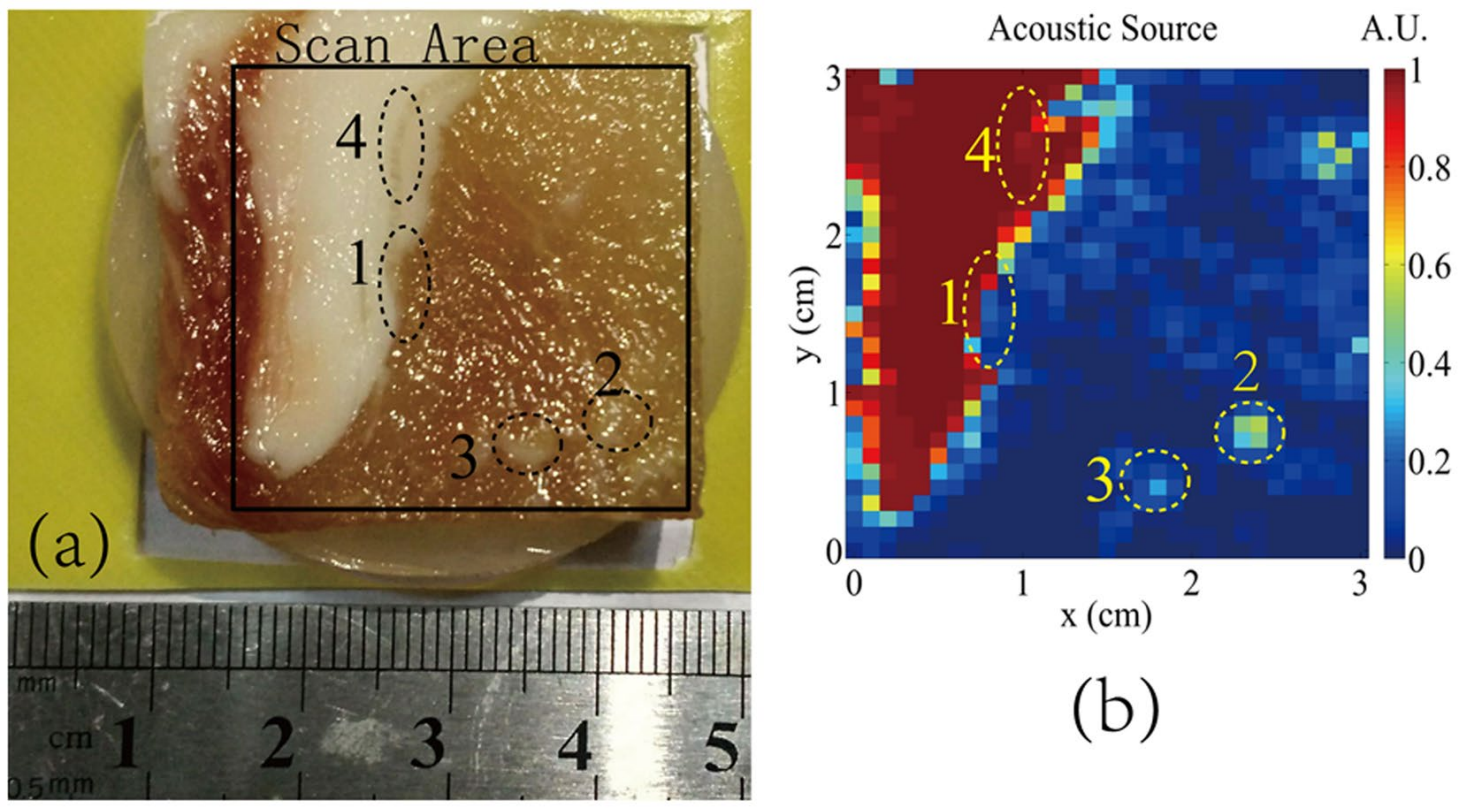
(**a**) Photograph of the fresh pork. (**b**) Reconstructed image of the thermo-acoustic source.

Considering the real tissues of complex geometry and inhomogeneous conductivity distribution, we used a sample of fresh pork to verify the imaging quality. The sample retained the natural formed fat-muscle structure as shown in Fig. 5(a). The size of the whole sample was 35 mm × 35 mm × 5 mm. Scanning of this phantom was done in a rectangle area of 30 mm × 30 mm with the step length of 1 mm along *x* and *y* directions. The real part of the resistivity was reconstructed as shown in Fig. 5(b). The reconstructed image was highly consistent with the original model with the high resolution and contrast. The high resolution of the image made it easily to recognize the part of the muscles that infiltrates into the fats and the part of the fats that infiltrates into the muscles, as shown in the area 1 and 4, respectively. Even some little fat distributed in the muscle could be imaged clearly as illustrated at the area 2 and 3.

## Discussion

If we move the plasma jet in scan mode, the real part of the complex resistivity can be imaged point by point under the assumption of the constant $\frac{j\omega \beta }{{C}_{p}}{|{\boldsymbol{J}}(\omega )|}^{2}$. The amplitude of the received signal indicates the intensity of the thermo-acoustic source near the contact point. On the other hand, if the excitation frequency of the conductive current can be changed, the real part of the resistivity at different frequencies could be reconstructed.

According to Eq. (6), if *σ* is equal to *ωε*, the acoustic source would take the maximum. When *σ* tends to 0 or infinity, the acoustic source would approach to 0. For biological tissues at the frequency of several Megahertz, the *ωε* is generally much smaller than *σ*, thus the acoustic source would increase with the conductivity decreases. That is to say, there are distinguished thermo-acoustic sources in ordinary biological tissues of low conductivity. Compared with the traditional thermo-acoustic excitation, such as MMTAT, the proposed method would provide high energy conversion efficiency between the electromagnetic excitation and the acoustic response.

It is worth mentioning that the length of the plasma jet should be kept constant for all scanning points in order to implement the constant pulsed current to the tissue. Therefore, the NEAPPJ should be fluctuated with the undulation of the tissue’s surface, unless the tissue has a flat surface without any ripple as well-designed in this experiment. For the general case, the length of the plasma jet can be measured by the propagating time of the thermo-acoustic signals if the distance between the tip of the tube 1 and the transducer is already known.

7 $${L}_{P}={L}_{T}-c\times {t}_{p}$$

Here *L*_P_ , *L*_T_, *c* and *t*_p_ represent the length of the plasma jet, the total length between the tip of the tube 1 and the transducer, the speed of sound and the propagating time of the thermo-acoustic signals, respectively.

The 200 ns pulsed conductive current from the plasma jet indicate the fundamental excitation frequency of 5 MHz. Considering the complex conductivity, the fat and muscle tissues exhibit the conductivities about 0.02 S/m and 0.4 S/m at this frequency, respectively. The thermo-acoustic signals from the fat would be more than ten times higher than that from the muscle, which leads to the high contrast of the image, as shown in Fig. 5. What’s more, the image resolution is determined by the diameter of the contact point. The smaller plasma jet and smaller step length of micrometer scale would further increase the resolution to distinguish finer conductivity changes. On the other hand, the penetration depth is limited by the spatial diffusion of the current density. An enlarged contact point would lead to the deeper penetration while lower resolution. A possible solution to this paradox lies in taking advantage of the different arrival time of the ultrasound to recognize the thermo-acoustic sources of different depths.

To conclude, the thermo-acoustic signal generation and imaging by the non-equilibrium atmospheric pressure plasma jet is demonstrated theoretically and experimentally. It can provide the resistivity imaging of the tissue’s surface with high contrast and resolution. With the same conductive current density, the thermo-acoustic source would take the maximum when the tissue’s conductivity *σ* is equal to *ωε*, which provides an approach to further improve the SNR by employing the proper frequency. Future works will aim to increase the penetration depth and the layering capability to demonstrate the significant 3-dimension imaging in biological tissues.

## Method

The experimental system setup along with its photograph is shown in Fig. 2. The quartz tube of the NEAPPJ device is fixed on the top of the oil tank. The unfocused ultrasonic transducer with center frequency of 1 MHz (V303, Olympus) is fixed on the bottom and immersed in the mineral oil to detect the thermo-acoustic signals. The sound speed of the mineral oil is calibrated as 1404 m/s. The quartz tubes, the transducer and the oil tank are all centered on the same *z* axis. The object stage is connected to a linear translation stage that can be moved in *x* or *y* directions. There is a rectangle hole on the object stage in order to transmit the ultrasound without attenuation while supporting the tissues. The NEAPPJ with the length of 1 cm and the diameter of about 1 mm as described before is employed to act on the target for the thermo-acoustic imaging. The NEAPPJ’s total current and the flow rate are monitored to ensure the constant conductive currents that are injected into the target. The target phantom is carried by the object stage and moved step by step to be scanned at different points, meanwhile the transducer and the NEAPPJ remain stationary. This optimized process would eliminate the influence from the directivity of the transducer. The acoustic signal is first amplified 80 dB with a customized two-stage low noise amplifier and then digitized by the oscilloscope (DSOX 2004A, Agilent). The data is averaged by the oscilloscope 16 times before being transferred to MATLAB on a PC for offline processing.
